# The Why, what, and How of the New FACT standards for immune effector cells

**DOI:** 10.1186/s40425-017-0239-0

**Published:** 2017-04-18

**Authors:** Marcela V. Maus, Sarah Nikiforow

**Affiliations:** 1 Cellular Immunotherapy Program, Massachusetts General Hospital Cancer Center, Boston, MA USA; 2 Department of Medicine, Harvard Medical School, Boston, MA USA; 3 Division of Hematologic Malignancies, Dana-Farber Cancer Institute, Boston, MA USA; 4 Harvard Medical School, Boston, MA USA

**Keywords:** Cellular therapy, Immune effector cells, Foundation for the Accreditation of Cellular Therapy, Chimeric antigen receptor T cells

## Abstract

Novel cellular therapies outside of traditional hematopoietic stem cell transplantation or hematopoietic progenitor cell (HPC) therapy are currently under evaluation in clinical trials across the United States and around the world. Several cellular products, e.g., CD19-directed Chimeric Antigen Receptor (CAR) T cells, are poised for FDA approval and thus increased use at a wider range of academic centers within the next year, with the likelihood of dissemination to standard oncology practice once safety is confirmed. However, these therapies entail some unique challenges in terms of logistics of delivery and toxicity management. Building on experiences and Standards established for HPC programs, the Foundation for the Accreditation of Cellular Therapy (FACT) has established new Standards specific to the use of Immune Effector Cells (IEC), including gene-modified T cells and natural (NK) cells. These Standards specify the clinical and quality infrastructure to facilitate safe administration of immune effector cells and formalize subsequent monitoring and reporting of patient outcomes to enable continual process improvement. Below we detail why these standards came into being, what they entail, and how a clinical team might access educational materials and implement these Standards. We propose that these Standards will be increasingly useful and relied up on as institutions and clinical service lines seek access to these treatment for their patients. FACT will begin accrediting programs that meet these new Standards for clinical administration of Immune Effector Cells in 2017.

## History and role of FACT

The Foundation for the Accreditation of Cellular Therapy (FACT) is a non-profit corporation co-founded in 1996 by the International Society for Cellular Therapy (ISCT) and the American Society of Blood and Marrow Transplantation (ASBMT) to provide a peer network of experts committed to improving stem cell transplantation and cellular therapy practices by formulating and disseminating evidence-based guidelines. These guidelines have until recently been formulated in 3 documents: 1) FACT Common Standards for Cellular Therapies, 2) FACT-JACIE Hematopoietic Cell Therapy Standards, and 3) NetCord-FACT Cord Blood Banking Standards [1–3]. These existing standards are applicable to general cellular therapy manufacturing and administration, to processing and clinical use of hematopoietic progenitor cells obtained from bone marrow or peripheral blood, and to umbilical and placental cord blood (UCB) banking, respectively. The Standards provide guidelines that span the entire spectrum of cellular therapy, from donor selection, to collection in an apheresis center or operating room, followed by simple or complex processing procedures, and then storage, shipping and labeling of the product, to finally administration and subsequent therapy-related care of the patient, including management and monitoring of toxicities and long-term outcomes.

In addition to providing training, educational activities, and guidelines which have been accepted in Europe, Canada, Australia, New Zealand and the US, FACT also offers a voluntary accreditation program for hematopoietic progenitor cell (HPC) therapy programs and UCB banks, with over 90% of eligible US HPC facilities and programs holding accreditation. Achieving FACT accreditation after a comprehensive inspection performed every 3 years demonstrates to patients, physicians, commercial manufacturers, regulatory agencies, and insurance payers that a given HPC program or UCB bank is committed to a certain level of quality measures and oversight in cell therapy practices and downstream patient care.

## Past, present, and future of adoptive immunotherapy, or “immune effector cells”

For the past 30 years or so, adoptive cellular therapy with immune effector cells such as tumor-infiltrating lymphocytes, ex vivo cultured lymphocytes, and genetically-modified lymphocytes have been developed at select academic medical centers. The resulting cellular products have generally been administered by clinical researchers in a variety of fields, including oncologic surgeons, medical oncologists, hematologists, and specialists in hematopoietic stem cell transplantation, under federal (FDA) and local regulatory (Institutional Review Board) supervision [4, 5]. Following demonstrations of the remarkable efficacy of genetically-modified T cells in certain hematologic malignancies, e.g., frequent and durable response rates in B cell malignancies treated with CD19-directed chimeric antigen receptor (CAR) T cell trials [6–11], the field has grown tremendously, with an explosion of academic and commercial investigations using immune effector cells as a new platform of therapy. Immune Effector Cells (IECs) currently include, but are not limited to, NK cells with or without ex vivo activation to exert broad cytotoxicity against tumor cells, genetically-modified T cells expressing CARs or engineered T cell receptors directed against tumor-associated antigens, cytotoxic T lymphocytes expanded ex vivo against viral or tumor peptides to target infection or malignancy, regulatory T cells with or without genetic modification to induce tolerance, and dendritic cells loaded with peptides or genetically engineered to express cytokines/chemokines in order to enhance immune recognition [12–14]. (Mesenchymal stem cells and other aspects of regenerative medicine and genetically-engineered tumor cell vaccines are also growing fields but are not considered IECs under the current Standards.)

As of December 15, 2016, there are currently 2219 open clinical trials of T cell-based cellular therapies and 265 NK-cell based cellular therapies around the world (clinicaltrials.gov) for many different types of cancer. These IECs are also being explored as treatment options in other clinical settings, such as solid organ transplantation and autoimmune disease. Whether these therapies are administered by “cellular therapists” derived from the HPC program, by disease-focused oncologists, or by physicians in other specialties is evolving independently at each clinical site. Many initial Phase I academic endeavors have led to commercial sponsorship and manufacturing to support larger Phase II and Phase III registration trials. To meet demand, commercial apheresis facilities may be called in to assist academic apheresis centers to allow participation of clinical sites without an internal collection facility. Thus, the number of physical entities and highly specialized individuals involved in collecting, manufacturing, delivering, and caring for patients receiving these types of therapies is likely to become greater than has historically been encountered by centralized transplant programs, and the experience base of the individuals involved in each step may be highly variable.

## Cellular therapies require special clinical infrastructure

The first round of regulatory approvals for IECs for cancer indications since the Provenge dendritic cell vaccine are expected in 2017. This will mark a significant milestone in the field, as CAR T cells become more widely commercially available outside of a research study environment. CAR T cells carry a more extensive toxicity profile than dendritic cell vaccines, namely the potential for tumor lysis; cytokine release syndrome requiring intensive unit care; neurologic toxicity ranging from encephalopathies characterized by aphasia, seizures, and in rare instances, fatal cerebral edema; as well as unusual immunologic manifestations such as hemophagocytic lymphohistiocytosis. As many investigators have learned, the administration of CAR-modified T cells can consume significant effort from various members of the healthcare team, including transfusion medicine colleagues, the cell-processing laboratory, pharmacy, outpatient and inpatient clinical teams, and specialty consultants. A robust clinical infrastructure is required to handle the complex scheduling logistics, maintain the chain-of-custody and chain-of-identity of the cellular product, and facilitate communication to manage potentially severe toxicities.

Many aspects of cellular therapy are different from established workflows in general oncology: i.e., cells are not stored in vials, nor are they mixed and re-labeled in the pharmacy. These products must be temperature-controlled at all times during preparation, shipping, and administration, and can only be manipulated under aseptic conditions. The importance of the chain-of-custody and absolute certainty regarding identity of the cellular product, along with its label and attendant paperwork, cannot be overstated, as administration of the wrong product can have lethal consequences. Furthermore, since IECs are “living drugs” their pharmacokinetics are less predictable than traditional small molecule drugs and even biologics such as antibodies. Toxicities can appear days after infusion of the cells and evolve rapidly. Both the outpatient and inpatient healthcare teams must be attuned to the potential toxicities, including cytokine release syndrome, to appropriately manage the patient. Clinical management may include administration of specialized drugs such as anti-cytokine monoclonal antibodies (i.e., tocilizumab), which need to be readily available to manage life-threatening toxicity but are not frequently used or necessarily available in routine healthcare [15, 16]. Finally, there are recommendations from regulatory agencies to monitor patients receiving genome-modified cellular products for up to 15 years to detect the emergence of potential long-term toxicities, such as secondary malignancies from insertional oncogenesis.

## FACT immune effector cell task force and generation of standards

Given these unique logistics and toxicity profiles, the need for novel supportive care and medications, and rapid progression of these technologies toward FDA licensing, academic cellular therapists and transplant programs expressed a desire to apply existing FACT standards and/or create directed guidelines to ensure similar safety measures in this new cellular therapy field. This impetus was seconded by many commercial cell manufacturers, regulatory agencies, and academic entities. With the help of a task force representing FACT, ISCT, American Society for Gene and Cellular Therapy (ASGCT), and Society for Immunotherapy of Cancer (SITC) leadership as well as academicians and cellular therapists from 10 cancer centers, FACT has formulated Standards and an Accreditation Program for IECs, defined as “cells used to modulate, elicit, or mitigate an immune response for therapeutic intent”, including dendritic, natural killer, T or B cells.

**Fig. 1:**
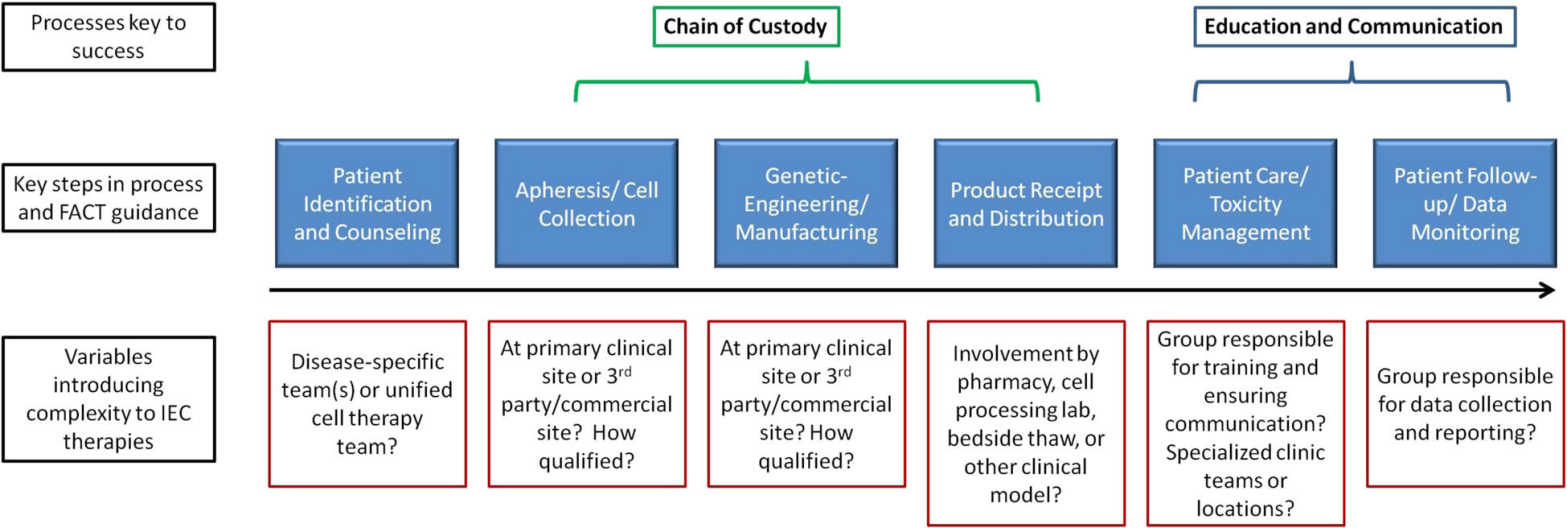
Complexities and crucial decisions/processes inherent in Immune Effector Cell delivery for which FACT standards offer guidance. IEC delivery will challenge a clinical program to determine which teams are involved in each step from patient identification to long-term follow-up (i.e., disease-specific, transplant, or a hybrid cell therapy team) and how to ensure communication and training flow smoothly between all clinical entities that may interact with a given patient. Specifically, with the introduction of “off-site” cell collection and/or manufacturing at other academic or commercial sites, a robust way to track chain of custody to ensure delivery to the correct patient is key

FACT Standards address processes, documentation and oversight, not the scientific validity, vector design or actual manufacturing steps for any given cell product. The FACT IEC task force, comprised of FACT medical staff and academic experts in the field, set out to review the primary guidelines present in the Common Standards, verify they were still appropriate, and then add requirements specific to IECs. In addition to standard recommendations for donor workup, apheresis collection, labeling, storage, documentation, and product administration, specific attention and guidance were given to 4 areas (Fig. 1): 1) Location of cell manufacturing: the level of involvement in manufacturing by a clinical site for a given IEC product may vary. Under FACT, programs are responsible only for the steps in which they are involved, e.g., donor workup, collection, and administration but potentially not the manufacturing of the cellular product if it occurs at a 3rd party or commercial laboratory. Despite site of manufacture, however, agreement on how to ensure and verify chain of custody through multiple handoffs from collection until infusion needs to be reached. Programs should ask for documentation of a quality audit or report to ensure that manufacturing by an unrelated party is taking place under appropriate regulatory oversight and following acceptable standards in the field of cellular therapy, bearing in mind that regulatory requirements are minimum requirements at best. 2) Identification and management of Cytokine release syndrome: specific medications and algorithms to manage this are still evolving. Therefore, the Standards do not suggest a specific management strategy but instead suggest that physicians, nurses and other providers at a clinical program have training to detect these complications and demonstrate competency in responding to them, that pharmacy formularies are adequate to treat anticipated toxicities, and that an institution have local guidance for management considerations for all the healthcare team members to access. 3) Coordination and education: given the multiple teams involved with a patient’s product and care, an institution should demonstrate appropriate communication pathways between the many providers involved and an avenue for rapid escalation of care when needed. 4) Data management and oversight: staff should be designated to collect data on product safety, efficacy and clinical outcomes, and these should be reviewed by the IEC program director at least yearly. Data points of interest are still being identified, but use of the CIBMTR Cellular Therapy forms is highly encouraged to allow pooling of data accessible to the entire field.

## Implementation of FACT IEC standards and caveats

**Fig. 2:**
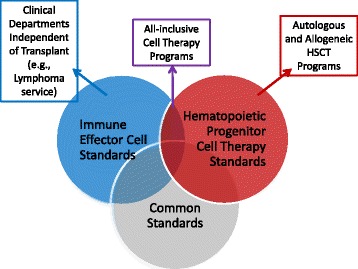
Relationship between different FACT standards. Individual programs will reference standards and seek accreditation as best suits their needs. While all cellular therapy manufacturing and distribution should comply with the guidelines in the Common Standards, clinical teams may operate and apply for accreditation within an HSCT program, an IEC program or one that involves both types of cell therapies

FACT recognizes that at any given clinical center, the clinical teams and infrastructure overseeing IECs versus HPCs may be different or present in isolation: i.e., there may be a stand-alone lymphoma IEC program at one site whereas a larger center may choose to merge IEC administration with their transplant program. For those reasons, accreditation for IECs is separate from that of HPC transplantation programs, although there is significant overlap in the guidance and infrastructure in the IEC and HPC standards (Fig. 2). At any center, the clinical teams administering cellular therapies will be expected to clarify which entities are applying for which type of accreditation and will need to demonstrate leadership, quality management and staff training programs that can be shared or unique. It is expected that inspections for IEC accreditation will start in the second half of 2017.

Given the rapid evolution of this field, it is anticipated that despite the incorporation of many comments from academic, professional society, and commercial entities during the public comment period, these Standards will remain a “work in progress”. Standards are being amended and finalized, and inspectors specific to IEC accreditation initiated specialized training at the recent 2016 meeting of the American Society of Hematology and the 2017 ASBMT/CIBMTR Tandem meeting Inspector information and links to the actual Standards can be found on the FACT website (www.FACTwebsite.org) and in references 1–3 below. FACT leadership is acting on requests to post educational material and templates for a quality assessment for third-party manufacturers. Toxicity management procedures with sample approaches are being formulated to provide potential approaches to programs less familiar with IEC administration and FACT processes in order to facilitate compliance with guidelines. Individual programs will seek accreditation as best suits their needs. US manufacturers and insurers will determine how FACT accreditation will factor into their decisions to release products or cover services at clinical sites. Additional regulatory oversight and reporting is required outside of the United States where these products fall into the ATMP (Advanced Therapy Medicinal Product) realm and must be handled according to European Medicines Agency. Currently, the FACT IEC standards represent expert consensus informed by many outside opinions as to basic expectations for quality and monitoring approaches in any program administering IECs. The hope is that the FACT IEC Standards will continue to serve as educational support and guidance for clinicians and regulators involved in these promising novel cellular approaches.

## Abbreviations

ASBMT: American Society of Blood and Marrow Transplantation
ASGCT: American Society for Gene and Cellular Therapy
ATMP: Advanced therapy medicinal product
CAR: Chimeric antigen receptor
CIBMTR: Center for International Blood and Marrow Transplant Research
FACT: Foundation for the Accreditation of Cellular Therapy
FDA: Food and Drug Administration
HPC: Human progenitor cell
IEC: Immune effector cell
ISCT: International Society for Cellular Therapy
NK: Natural Killer cell
SITC: Society for Immunotherapy of Cancer
UCB: Umbilical cord blood
US: United States of America
